# U-shaped link of health checkup data and need for care using a time-dependent cox regression model with a restricted cubic spline

**DOI:** 10.1038/s41598-023-33865-x

**Published:** 2023-05-22

**Authors:** Masahiro Nakatochi, Akitaka Sugishita, Chihiro Watanabe, Etsuko Fuchita, Masaaki Mizuno

**Affiliations:** 1 Public Health Informatics Unit, Department of Integrated Health Sciences, Nagoya University Graduate School of Medicine, Nagoya, 461-8673 Japan; 2 Department of Advanced Medicine, Nagoya University Hospital, Nagoya, 466-8560 Japan; 3 Department of Nursing, Nagoya University School of Health Sciences, Nagoya, 461-8673 Japan; 4 Gerontological Nursing, Department of Integrated Health Sciences, Nagoya University Graduate School of Medicine, Nagoya, 461-8673 Japan; 5 Gerontological Nursing, Faculty of Nursing, Department of Nursing, Kawasaki City College of Nursing, Kawasaki, 212-0054 Japan

**Keywords:** Geriatrics, Public health

## Abstract

We explored risk indicators likely to result in older adults needing certified long-term care in Japan and ascertained whether this relationship forms a U-shaped link. We analyzed a community-based cohort of residents in Kitanagoya City, Aichi Prefecture, Japan. Participants were 3718 individuals aged 65 years and above who underwent health examinations between April 1, 2011 and March 31, 2012. For continuous clinical variables, we applied a time-dependent Cox regression model. Two types of models were applied—a linear and nonlinear model with restricted cubic splines—to assess the U-shaped association. Statistical significance (set at 0.05) for the nonlinearity was tested by comparing the spline and linear models. Among the participants, 701 were certified as needing Level 1 care or higher during a follow-up. Among the continuous clinical variables, the nonlinear model for body mass index, systolic blood pressure, high-density lipoprotein cholesterol, alanine aminotransferase, aspartate aminotransferase, and γ-glutamyl transpeptidase revealed significant U-shaped associations as compared with the linear model in which the outcome was a certification of the need for nursing care. These results provide an important insight into the usefulness of nonlinear models for predicting the risk of such certification.

## Introduction

Global trends show a steady rise in the aging rate of the adult population aged 65 and above, which will likely continue in the coming decades. Japan alone had 36.19 million older adults by 2020, nearly 28.8% of its total population^1^, making it a super-aging society. The National Institute of Population and Social Security Research predicts that the older adult population in Japan will peak at 39.35 million in 2042; by 2036, 1 out of 3 people (33.3%), and by 2065, 1 out of 2.6 people (38.4%) will be older adults^2^.

Aging is a dynamic process. As the demographic of older adults increases, so will the need to provide this population with appropriate nursing care. In response to this social evolution, the Japanese government introduced long-term care insurance in 2000 to replace the obsolete welfare system^3^. The new system is a type of long-term social insurance that prioritizes the independence of older adults and offers a greater choice of services for beneficiaries. This has made it popular for older adults who demand greater home care to maintain their quality of life^4^.

To become a beneficiary under the long-term care insurance system, an individual must apply for a long-term care (support) certification and undergo an assessment to gauge the level of service required. The assessment follows nationally uniform certification standards for those requiring long-term care. Applicants are classified as those requiring support (Support Level 1 or 2), those requiring long-term care (Care Need Levels 1 to 5), or those not requiring such support or care^5^. Older adults assessed as Support Level 1 and Support Level 2 are independent but need partial support. Care Need 1 is a state in which the patient has some limitations in activities of daily living, but is independent in basic activities of daily living, and Care Need 2 requires partial intervention owing to a decline in daily living activities. Care Need 3 is a state in which the patient's abilities decline for both types of activities, and full nursing care is required. Care Need 4 is a state in which the patient's mobility is low and assistance is necessary. Finally, Care Need 5 is a state in which the patient's mobility is very low and daily living without assistance and care is impossible.

In 2019, 4.8 million people of all ages (95% over 65) were certified as requiring nursing care—2.1 times the number in 2000^6^. Fifty percent of long-term care insurance costs are covered by public funds, and the balance is covered by premiums paid by the insured^7^. Insurance premiums for people aged 65 or older doubled between 2000 and 2021^8^. As the older adult population is expected to increase further, the government must devise better policies—both healthcare and financial—for new beneficiaries. One solution, albeit difficult, is to identify people at high risk of becoming certified for long-term care and extend healthy life expectancy through preventive health interventions. Such a solution requires us to find appropriate and sensitive risk markers to identify high-risk individuals.

According to the 2019 National Survey of People’s Lives, dementia (17.6%), cerebrovascular disease (16.1%), and age-related weakness (12.8%) are the most common causes for care needs among those requiring Level 1 assistance or more^9^. Joint disease, dementia, and cerebrovascular disease were the most common causes among those requiring support, Level 1–3 care, and Level 4–5 care, respectively. To prevent the need for nursing care at an earlier stage, identifying markers for these diseases before they progress to a point where intervention is required may be necessary. Meanwhile, long-term care is required for various reasons. Therefore, there is a need for uniform risk markers rather than disease-specific ones. The Japanese government mandates that all individuals between 40 and 74 years with medical insurance receive a specified health checkup every year to prevent lifestyle disorders, particularly metabolic syndrome. High-risk individuals are provided guidance and support by healthcare staff to improve their lifestyle as a preventative measure^10^. In Aichi Prefecture, the Aichi Prefecture Wide-Area Union for Late-Stage Senior Citizen’s Health Care commissions each municipality to conduct health examinations for older adults who are 75 years and above^11^. If the risk factors or markers that lead to the identification of care needs can be determined from these health checkup data, and if risk prediction for the need for nursing care can be implemented, more rapid and precise interventions can be developed to delay or prevent the need for nursing care.

Research on discovering risk factors for certified long-term care needs is abundant, with many studies on the Japanese population using health checkup data for this purpose^12–15^. However, these data are derived from the start of the follow-up, whereas information assessed in health examinations, such as body mass index (BMI), can change over time. As most Japanese people undergo an annual health checkup, any exploration of risk should involve the monitoring of longer-term health examination data. Many studies use linear models to evaluate the relationship between risk factors and outcomes. However, risk factors and outcomes may show a nonlinear relationship. Studies note that high and low BMI and other clinical parameters raise the risk of death and disease development^16–18^. Large-scale studies and meta-analyses propose a U-shaped relationship between adult BMI and all-cause mortality^19–23^. When continuous clinical variables are disproportional to the risk of not only death and development of diseases but also certified long-term care, a nonlinear model should be used for evaluation rather than the commonly used linear model. Restricted cubic spline models, which we employ in our analysis, are often used to examine nonlinearities in detail^22^.

In this study, we employed 8.5 years of follow-up data and 9 years of health checkup data of older adults enrolled under long-term care insurance in Kitanagoya City, Japan. We explored risk indicators, which are risk factors and risk markers, likely to result in older adults needing certified long-term care, using a time-dependent Cox analysis, which also considers nonlinear models. Mortality has been previously evaluated in relation to various clinical parameters^20, 24^. Therefore, to evaluate the validity of this analytical approach, we also conducted the analysis with death as the outcome.

## Methods

### Study design

**Figure 1:**
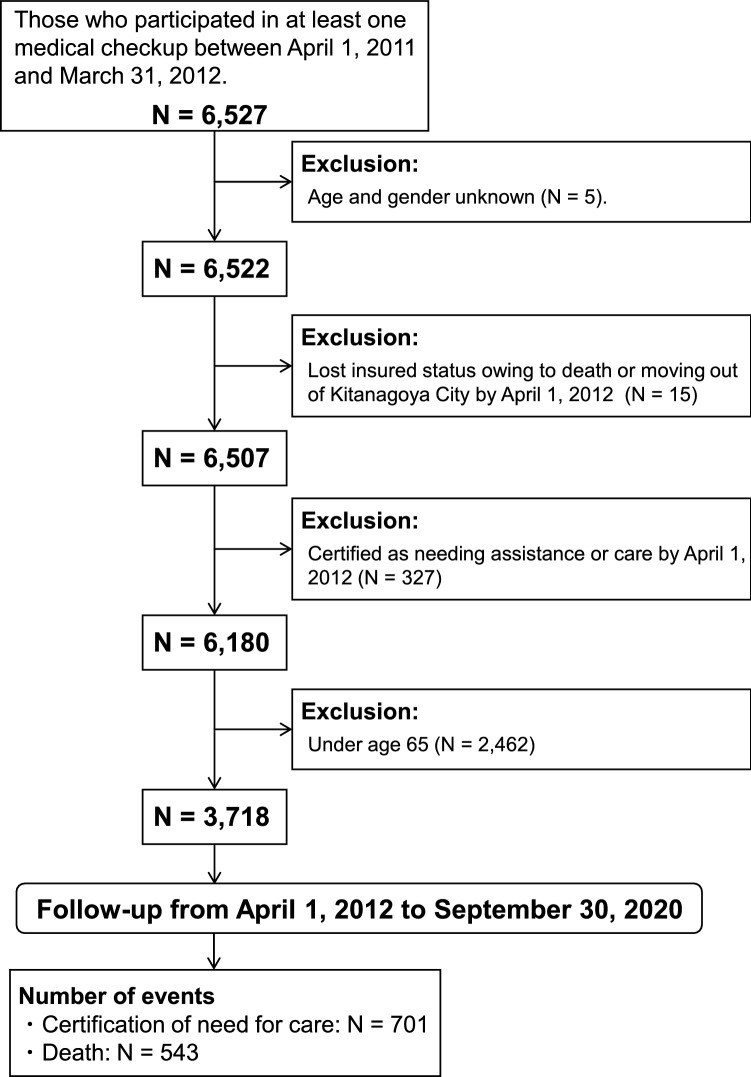
Flowchart of subjects.

We employed a community-based retrospective cohort study to explore the risk indicators for certified long-term care needs and mortality among older adults. We integrated and analyzed two sets of data received from Kitanagoya City, Aichi Prefecture, Japan. The first dataset comprises long-term care information on certification, mortality, and transfers out of Kitanagoya City from April 2011 to September 2020. The second dataset is health checkup data comprising specific health checkup information and late-stage health checkup data from 2011 to 2019. Figure 1 shows the selection of the study participants. Using the second dataset, we first confirmed that 6527 residents of Kitanagoya City in Aichi Prefecture participated in at least one health checkup between April 1, 2011 and March 31, 2012. Then, we applied the following exclusion criteria to the 6527 participants: (1) age and gender unknown; (2) lost insured status owing to death or moving out of Kitanagoya City by April 1, 2012; (3) certified as needing assistance or care by April 1, 2012; and (4) under age 65. The final sample included 3,718 persons aged 65 years or above as of April 1, 2012, who had undergone health examinations between April 1, 2011 and March 31, 2012. Participants were eligible for follow-up from April 1, 2012 to September 30, 2020. Those newly certified as needing long-term care and those who had died were identified from the first dataset.

The study protocol adhered to the guidelines of the Declaration of Helsinki. This study was approved by the Ethics Review Committee of Nagoya University Graduate School of Medicine (No. 2019-0100) and was conducted under contract with Kitanagoya City, Aichi Prefecture. As the data used in this study were anonymized and non-personalized by Kitanagoya City, Aichi Prefecture, which is the data management authority, the participants’ informed consent is not necessary based on the “Ethical Guidelines for Medical and Health Research Involving Human Subjects” in Japan^25^.

### Outcome variables

In this study, the outcome was whether the older adult was certified as requiring Level 1 or higher nursing care, or had died. Follow-up was terminated if the participant had died or moved out of the city, or was certified as needing long-term care. The outcome variables were obtained as time-to-event data.

To use the long-term care insurance system, one must apply for a long-term care (support) certification and undergo a two-stage assessment to determine the level of care required. The first is a computerized examination of 74 survey items (primary assessment), followed by an examination (secondary assessment) by the Long-Term Care Certification Examination Board established by the municipality based on the primary assessment results, the attending physician's written opinion, and other materials^5^. We focused only on the certification of the need for long-term care (Care Need Levels 1 to 5). If the participant was certified as requiring Level 1 or higher care in the secondary assessment, the date of this secondary assessment was used as the date of certification.

As the death data dates obtained from Kitanagoya City were monthly, the last day of the month was used as the date of death.

### Health checkup data

The health checkup data consist of specific medical checkup and late-stage health checkup information from 2011 to 2019. Of the health checkup items, those items consistently obtained for all participants in all years were included in the analysis. The health checkup data include basic attributes, test items of the specific health checkups and health checkups for specific older adults, and the participant’s questionnaire responses. The basic attributes include age and gender. Anthropometric information includes height, weight, and BMI. Clinical laboratory information includes blood pressure (systolic blood pressure or SBP and diastolic blood pressure or DBP), alanine aminotransferase (ALT), aspartate aminotransferase (AST), γ-glutamyl transpeptidase (γ-GTP), HbA1c content, and serum lipid profile. Tests for glucose and protein in urine were also performed. HbA1c was measured by the Japanese Diabetes Society until 2012 and by the National Glycohemoglobin Standardization Program from 2013. The National Glycohemoglobin Standardization Program values were converted to Japanese Diabetes Society values and used for further analysis. Information on smoking status, drinking status, and disease history was obtained through a questionnaire.

Diabetes mellitus, hypertension, and dyslipidemia were defined, respectively, as (1) HbA1c of ≥ 6.1%, or treatment with blood glucose-lowering drugs; (2) SBP of ≥ 140 mmHg, DBP of ≥ 90 mmHg, or treatment with antihypertensive drugs; and (3) high-density lipoprotein cholesterol (HDL-cho) concentration of < 40 mg/dL, low-density lipoprotein cholesterol (LDL-cho) concentration of ≥ 140 mg/dL, triglyceride concentration of ≥ 150 mg/dL, or treatment with antidyslipidemic drugs.

### Statistical analyses

The distributions of continuous variables at the baseline were compared between men and women participants using the Student’s *t*-test or Wilcoxon rank sum test. The distributions of categorical variables at the baseline were compared between men and women using Fisher’s exact test.

Crude rates of certified care needs and death are shown as the number of certified care need cases and deaths per 1,000 person-years, respectively. The crude rates were compared between men and women using the Wald test.

For continuous clinical variables, a time-dependent Cox regression analysis^26^ was performed to assess the association of each clinical parameter with certified care need and death, using data measured at the baseline and over several years of follow-up. For the Cox regression, we used the time to the first certification of need for care or the time to death as time-to-event data. The level of care required was not considered in the analysis of nursing care needs. Gender and age were included in the model as potential confounders. Furthermore, the same analysis was performed by adding treatment with blood glucose-lowering drugs (yes = 1, no = 0), treatment with antidyslipidemic drugs (yes = 1, no = 0), treatment with antihypertensive drugs (yes = 1, no = 0), smoking status (habitual smoker: yes = 1 vs. no = 0), and drinking status (habitual or chance drinker = 1 vs. non-drinker = 0) to the model as potential confounders, in addition to age and gender. Given that the distribution of triglyceride, ALT, AST, γ-GTP, and HbA1c levels was skewed, the values were log_2_-transformed (Supplementary Fig. S1). Cox regression analysis was compared by building both linear and nonlinear models. In the case of the nonlinear model, a restricted cubic spline was applied. Details of the analysis conditions are described in Supplementary Methods. Statistical significance in the nonlinear model was tested by comparing the spline and null models, while that for nonlinearity was tested by comparing the spline and linear models. A *p*-value < 0.05 for the test of nonlinearity and a *p*-value < 0.05 for the spline model depict a statistically significant nonlinear relationship between the clinical parameter and the event, and a spline model is adopted. Conversely, a linear model is adopted for a *p*-value ≥ 0.05 for the test of nonlinearity and a *p*-value < 0.05 for the linear model. Values of the Akaike information criterion (AIC) were compared between linear and nonlinear models to assess the quality of each model.

For categorical clinical variables, we performed a time-dependent Cox regression model to estimate hazard ratios (HRs) and 95% confidence intervals (CIs) for the association of each clinical variable with a certified need for long-term care and death data, measured at the baseline and over several years of follow-up. One of the categories—for each categorical parameter—was used as the reference category and the HR for the remaining categories was estimated. Gender and age were included in the model as potential confounders.

All statistical analyses were performed using R version 3.6 (http://www.r-project.org/). A *p*-value under 0.05 was considered significant. We used the survival package^27^ and the rms package^28^.

## Results

### Characteristics at the baseline

The follow-up participants included 3,718 individuals (1742 men; 1976 women) aged 65–97 years at the baseline. They participated in the specified health checkup or late-stage health checkup in Kitanagoya City between April 1, 2011 and March 31, 2012. The baseline backgrounds for the continuous and categorical variables of participants are shown in Table 1 and Supplementary Table S1, respectively. The mean age (SD) at the beginning of the follow-up was 73.1 (5.2) for men and 73.0 (5.5) for women. No significant gender differences in mean age at the baseline were identified.

**Table 1:** Characteristics of participants for continuous variables at the baseline.

Variable	Male (N = 1742)	Female (N = 1976)	*p*-value*
Age (years)	73.1 (5.2)	73 (5.5)	0.479
Height (cm)	162.5 (5.9)	149.9 (5.8)	< 0.001
Weight (kg)	61.3 (8.8)	50.9 (8.1)	< 0.001
BMI (kg/m^2^)	23.2 (2.8)	22.6 (3.3)	< 0.001
SBP (mmHg)	132.2 (17.6)	132.5 (18.4)	0.629
DBP (mmHg)	75.4 (11.4)	74 (11.2)	< 0.001
HDL-cho (mg/dL)	54.5 (14.3)	63.3 (15.5)	< 0.001
LDL-cho (mg/dL)	117 (29.2)	126.3 (29.4)	< 0.001
Triglyceride (mg/dL)	110 (81–160)	102 (76–142)	< 0.001
AST (IU/L)	23 (19–28)	22 (19–26)	< 0.001
ALT (IU/L)	18 (14–25)	16 (13–21)	< 0.001
γ-GTP (IU/L)	28 (20–44)	18 (15–26)	< 0.001
HbA1c (%)	5.3 (5.0–5.6)	5.3 (5.0–5.6)	0.376

Continuous data are means (SD) or medians (25–75 percentile). *BMI* body mass index, *SBP* systolic blood pressure, *DBP* diastolic blood pressure, *HDL-cho* high-density lipoprotein cholesterol, *LDL-cho* low-density lipoprotein cholesterol, *AST* aspartate aminotransferase, *ALT* alanine aminotransferase, *γ-GTP* γ-glutamyl transpeptidase.

*The *p*-values were calculated using Student’s t-test and Wilcoxon’s rank sum test.

### Crude event rates for care needs certification and death

The 3,718 participants in this study were followed for up to 8.50 years from April 1, 2012 to September 30, 2020. The mean follow-up was 7.46 years for certified care needs and 7.90 years for the event of death. The mean value of participation in health checkups per person was 6.08 when the event was certification and 6.17 when the event was death. Table 2 shows the number of subjects with events and details of the first certification of needing long-term care. Among participants, 701 (335 men, 26.2 per 1000 person-years; 366 women, 24.5 per 1000 person-years) were certified as needing Level 1 care or higher during the follow-up period. During the follow-up period, 543 subjects (340 men, 25.2 per 1000 person-years; 203 women, 12.8 per 1000 person-years) died. Mortality rates were significantly higher for men than for women (*p*-value < 0.001).

**Table 2:** Incidence rate of the need for care and all-cause death.

Variable	Category	Incidence per 1000 person-years		*p*-value*
		Male	Female	
Care need		26.2	24.5	0.384
Initial level of care need when first certified as requiring long-term care	Care level 1	11.9	15.3	0.014
	Care level 2	5.5	4.6	0.281
	Care level 3	4.1	1.8	< 0.001
	Care level 4	3.0	1.8	0.046
	Care level 5	1.8	1.0	0.075
All-cause death		25.2	12.8	< 0.001

*The *p*-value was calculated using the Wald test.

### Association of each continuous parameter with the events

**Figure 2:**
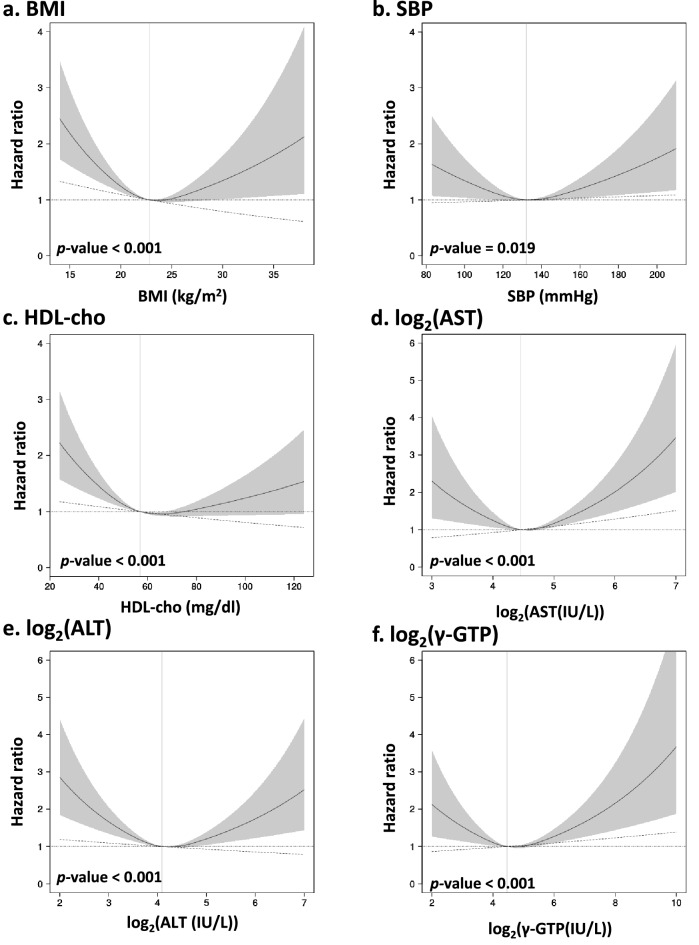
Hazard ratios of certified need for care estimated by a time-dependent Cox model with a restricted cubic spline model (three knots). The black straight line indicates the hazard ratio (HR) estimated for the spline model. The gray area represents the 95% confidence intervals of HR for the spline model. The vertical gray straight line indicates the reference value for the clinical parameter. The reference value was calculated as a median value of the clinical parameter. The dot-dash line indicates the HR estimated for the linear model. HRs are estimated when age is fixed at median age at baseline and gender is fixed at female. The *p*-values shown in the figures were calculated by the likelihood ratio test of the spline model against the null model. All *p*-values for nonlinearity were less than 0.05. *BMI* body mass index, *SBP* systolic blood pressure, *HDL-cho* high-density lipoprotein cholesterol, *AST* aspartate aminotransferase, *ALT* alanine aminotransferase, *γ-GTP* γ-glutamyl transpeptidase.

**Figure 3:**
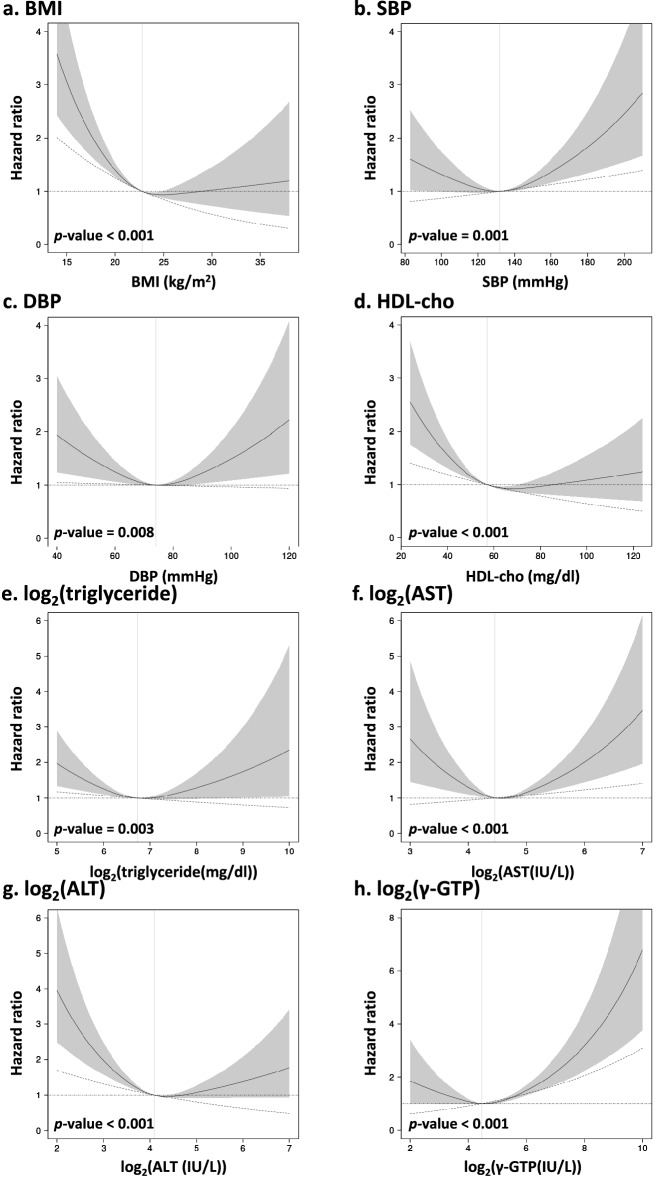
Hazard ratios of all-cause death estimated by a time-dependent Cox model with a restricted cubic spline model (three knots). The black straight line indicates the hazard ratio (HR) estimated for the spline model. The gray area represents the 95% confidence intervals of HR for the spline model. The vertical gray straight line indicates the reference value for the clinical parameter. The reference value was calculated as a median value of the clinical parameter. The dot-dash line indicates the HR estimated for the linear model. HRs are estimated when age is fixed at median age at baseline and gender is fixed at female. The *p*-values shown in the figures were calculated by the likelihood ratio test of the spline model against the null model. All *p*-values for nonlinearity were less than 0.05. *BMI* body mass index, *SBP* systolic blood pressure, *DBP* diastolic blood pressure, *HDL-cho* high-density lipoprotein cholesterol, *AST* aspartate aminotransferase, *ALT* alanine aminotransferase, *γ-GTP* γ-glutamyl transpeptidase.

For continuous clinical variables, we performed a time-dependent Cox regression model to estimate HRs and 95% CIs for the association of each clinical parameter with certification for long-term care and death. Two types of models were applied: a linear model and a nonlinear model with restricted cubic splines. Both models were compared using nonlinearity tests, and each of the two models was further compared with the null model. As a result, it was determined that the spline model was valid for BMI, SBP, HDL-cho, AST, ALT, and γ-GTP in terms of the certification of need for care (Supplementary Table S2). Similarly, for death, the spline model was determined as valid for BMI, SBP, DBP, HDL-cho, triglycerides, AST, ALT, and γ-GTP (Supplementary Table S3). The AIC value of the spline model for each of these variables was lower than the corresponding ones of the linear model. HRs estimated by spline models for other continuous variables are shown in Figs. 2 and 3. These variables indicate that both low and high values increase the risk of certification of need for care and death, respectively.

The analysis showed that DBP and triglycerides were not significantly associated with the risk of needing care compared to the null model for the spline and linear models (Supplementary Table S2). The linear model was deemed valid for LDL-cho and HbA1c in terms of the certification of need for care (Supplementary Table S2). The linear model was deemed valid for LDL-cho in terms of death (Supplementary Table S3). Higher LDL-cho was associated with a significantly lower risk of certification and death. Higher HbA1c was associated with an increased risk of certification. Departures from the proportional hazard were checked for each clinical parameter, using Schoenfeld residuals for selected spline and linear models. We found no significant departure from proportional hazards in any of the models (Supplementary Figs. S2 and S3).

Furthermore, the same analysis was performed by adding treatment, smoking status, and drinking status to the Cox model as adjustment factors, in addition to age and gender. The results were similar to those without the additional variables (Supplementary Tables S4 and S5, Supplementary Figs. S4–S7).

### Association of each categorical parameter with the events

The results of the association analysis of each categorical clinical parameter with the events are shown in Table 3. In the case of urinalysis, the risk of needing nursing care and death was significantly higher for those who did not have negative urine glucose and/or negative urine protein than for those who did.

**Table 3:** Hazard ratio of the clinical categorical parameter for each outcome.

Variable	Category	Reference	Care need		All-cause death	
			HR (95% CI)	*p*-value*	HR (95% CI)	*p*-value*
Urinalysis						
Urine glucose	±, +, ++, +++	–	1.60 (1.20–2.15)	0.003	1.59 (1.16–2.17)	0.007
Urine protein	±, +, ++, +++	–	1.51 (1.28–1.78)	< 0.001	1.59 (1.33–1.91)	< 0.001
History of disorder						
Undergoing treatment with blood glucose-lowering drugs	Yes	No	1.20 (0.94–1.52)	0.152	1.16 (0.88–1.52)	0.297
Undergoing treatment with antidyslipidemic drugs	Yes	No	0.76 (0.64–0.90)	0.001	0.61 (0.50–0.75)	< 0.001
Undergoing treatment with antihypertensive drugs	Yes	No	1.04 (0.90–1.21)	0.589	0.92 (0.78–1.10)	0.365
History of stroke	Yes	No	1.64 (1.27–2.12)	< 0.001	1.58 (1.21–2.06)	0.002
History of heart disease	Yes	No	1.41 (1.16–1.72)	0.001	1.32 (1.06–1.66)	0.018
History of renal failure	Yes	No	1.89 (1.01–3.53)	0.071	2.08 (1.11–3.90)	0.040
History of anemia	Yes	No	1.18 (0.93–1.49)	0.181	1.39 (1.08–1.79)	0.014
Disorder						
Underweight	Yes	No	1.56 (1.26–1.93)	< 0.001	1.87 (1.48–2.36)	< 0.001
Overweight or obese	Yes	No	1.02 (0.85–1.23)	0.808	0.80 (0.64–0.99)	0.040
Diabetes	Yes	No	1.38 (1.14–1.67)	0.002	1.05 (0.83–1.32)	0.699
Hypertension	Yes	No	1.11 (0.94–1.31)	0.205	1.03 (0.86–1.24)	0.724
Dyslipidemia	Yes	No	0.83 (0.71–0.96)	0.013	0.69 (0.58–0.82)	< 0.001
Lifestyle						
Habitual smoker	Yes	No	0.91 (0.66–1.24)	0.531	1.25 (0.94–1.66)	0.142
Drinker	Habitual, chance	Non	0.79 (0.66–0.93)	0.006	0.75 (0.62–0.91)	0.003

The hazard ratio (HR) values represent the increased risk of each outcome in the other category against the reference. *CI* confidence interval.

*The *p*-values were calculated using the likelihood ratio test.

For disease history, participants on medications for lipids had a significantly lower risk of needing care and of death than those not on medications. Those with a history of stroke or heart disease had a significantly higher risk of needing long-term care certification and of death than those without. Those with renal failure and insomnia had a significantly higher risk of death.

For disorders, the underweight group had a significantly higher risk of needing long-term care certification and of death than the other groups. Those who were overweight or obese had a significantly lower risk of death than those who were not. People with diabetes were at significantly higher risk of needing long-term care certification. Further, those with dyslipidemia had a significantly lower risk of needing long-term care and of death.

## Discussion

While several studies have attempted to reveal risk factors for the certification of long-term care needs from health examination data of the Japanese population^12–15^, they have used only health examination data at the baseline. We performed a time-dependent Cox regression analysis using all health examination data at the baseline and during follow-ups. Time-dependent covariates may be a powerful tool for exploring predictive relationships by using quantities that vary over time^26^.

When applying continuous value variables to regression models, it was common to use only linear models in which the variable is inputted as an independent variable. However, linearity should not be assumed without evaluating that the association is indeed linear^29–31^. If the linearity assumption is disregarded and the association is nonetheless estimated to be linear, the effect estimates do not represent the true underlying effect and may introduce bias. To ensure unbiased effects estimates, nonlinear associations need to be modeled explicitly. Failure to estimate truly nonlinear relationships as nonlinear may lead to overestimation or underestimation of exposure effects^31^. The use of regression models with dummy variables and categorization of the continuous variable by criteria values are common. However, the categorization has multiple problems, including loss of information, discontinuities in estimated mean outcome values when moving from one category to another, and difficulty in comparing results across studies because cutoff points may be data-dependent^28, 31–35^. A U-shaped or reverse J-shaped association between BMI and death has been reported^19–23^. BMI may also have a U-shaped association with the risk of needing long-term care, and a similar association may be confirmed for other clinical parameters. Therefore, in this study, a restricted cubic spline model was considered to evaluate U-shaped or reverse J-shaped associations in addition to the linear model.

The present study confirms the significant U-shaped association between BMI and death (Fig. 3a), as previously reported^19–23^. We also observed a U-shaped association between BMI and the risk of needing long-term care certification (Fig. 2a). Studies also report that low body weight with a BMI < 18.5 is a risk factor for certified nursing care in Japan^12, 14^, and low body weight with a BMI < 20 is a risk factor for dementia in the United Kingdom^36^. Meanwhile, a high BMI raises the risk of developing cardiovascular disease, which is one of the main causes of the certification of care needs. Zhang et al. observe a U-shaped relationship between BMI and all-cause disability, with the risk of disability being significantly higher for participants with lower and higher BMIs in Japan^37^. Our results and these reports indicate that our approach of applying a spline model to examine U-shaped associations with BMI and other clinical parameters is appropriate for this study. If the association between risk and other clinical parameters is U-shaped rather than linear, more accurate estimates than previously reported hazard ratios are expected.

Our results suggest significant U-shaped associations of SBP, HDL-cho, AST, ALT, and γ-GTP with the risk of both needing care and death. However, DBP and triglyceride were found to have a significant U-shaped association with death. These results were similar when treatment status, smoking habits, and drinking habits were added as adjustment factors. These variables are known to be associated with cardiovascular disease and dementia. In Japan, cerebrovascular disease and dementia are one of the major causes of long-term care needs^9^. Our study also showed that a history of stroke was significantly associated with the need for care. Therefore, the U-shaped association with the risk of needing care identified in this study may have been a combined association with these diseases. Dementia and cerebrovascular disease are the leading factors in the certification of long-term care, but other factors also exist^9^. This study reveals an average U-shaped association between these clinical parameters and the risk of certified need for care due to various factors. Regarding needing care, DBP and triglycerides were significantly nonlinear in the comparison of linear and spline models; we did not observe any significant association with the risk of needing care in the comparison of spline and null models. The results indicate insufficient power to assess DBP and triglycerides; more samples are needed for the evaluation of these variables.

Liver enzymes such as AST, ALT, and γ-GTP also showed a U-shaped association with certified care and risk of death. Japanese patients with high levels of liver function enzymes have a higher risk of needing nursing care, but the risk of having low levels has not been evaluated^12, 13^. Our study is the first to report that low levels of these enzymes are also associated with high risk. Liver enzymes reveal a U-shaped association with the risk of developing cardiovascular disease, mortality, and cancer mortality^18, 38, 39^. Low levels of liver enzymes pose a risk of causing dementia^40^. Our results are expected to reflect the combined association with these diseases.

For LDL-cho and HbA1c, we found no significant improvement in the spline model over the linear model. This implies that a simpler linear association between LDL-cho/HbA1c and outcome events is reasonable. In fact, lower LDL-cho increased the risk of needing care. This result is inconsistent with the finding that elevated LDL-cho increases the risk of cardiovascular disease^41^. However, the results are consistent with those of Tsuji et al.^14^ who found that the group with LDL-cho of 140 mg/dL or higher had a lower risk of needing assistance and certification for long-term care (HR 0.85) than the group with LDL-cho less than 140 mg/dL in the Japanese population. These results are consistent when adjusted for lipid treatment status and other confounders. In our study, participants receiving lipid-lowering treatment had a lower risk of needing long-term care certification than those who did not. It is possible that those with LDL-cho high enough to require treatment may have lowered their LDL-cho levels with treatment, which is why no significant U-shaped association between LDL-cho and risk certification and death was observed.

Diabetes affects the entire body, including the nervous, retinal, renal, cardiovascular, and cerebrovascular systems. Older adults with persistently high blood glucose levels require nursing care as they are likely to develop further health complications. In this study, higher HbA1c was shown to increase the risk of certification for care requirements; diabetes also showed a higher risk (HR 1.38). Patients with diabetes are known to have a higher risk of developing dementia than their healthy counterparts^42^. There have long been reports that diabetes increases the risk of needing long-term care^15, 43^. The finding that diabetes is a risk factor for the certification of the need for long-term care is consistent with these reports. Nevertheless, this study is the first to report a linear association between HbA1c and the risk of needing long-term care certification.

This study has some limitations. First, this is a cohort study of a single region, pointing to the need to replicate our results in other regional populations. The certification of the level of care required is a program unique to Japan, and these results are not directly applicable to other countries. Second, this is an observational study where, in addition to models that considered only age and gender as adjustment factors, models adjusted for age, gender, treatment, smoking, and alcohol consumption were also analyzed to confirm the consistency of the results. Besides these factors, some potential covariates confound the association of interest, and their effects were not adjusted, which is a limitation of this study. For example, the systematic review by Tanaka et al. describes physical conditions (e.g., motor function, physical frailty, sarcopenia) and social factors (social participation, social support, social capital, and social frailty) as some of the most frequently reported risk factors for the certification of need for long-term care^44^. These items related to physical conditions and social factors are not obtained in general health checkups in Japan and were also not obtained in this study. Future studies could confirm whether these physical conditions and social factors contribute independently to the items found to be significantly associated with the level of care needed in this study, or to assess whether the prediction accuracy can be further improved by considering physical condition and social factor-related items. However, it is important to have markers that can predict the risk of obtaining a certification of need for long-term care from items obtained in existing general health checkups. The items found to be significantly associated with the certification of need for long-term care in this study were obtained from general health checkups and will be useful as risk markers for predicting the need for certification for long-term care even when these physical conditions and social factors are not available. Third, participation in specific health checkups and late-stage health checkups was not mandatory. It is possible that those who participated in health checkups are more health-conscious, which points to a selection bias.

## Conclusions

In conclusion, our results revealed a significant U-shaped association of BMI, SBP, DBP, HDL-cho, triglyceride, AST, ALT, and γ-GTP with the risk of being certified for long-term nursing care and/or of death. The results revealed a significant linear association of LDL-cho and HbA1c with the risk of being certified for long-term nursing care and of death. These results yield more precise estimates than those obtained by conventional categorization and provide an important insight into the usefulness of nonlinear models for predicting the risk of such certification. Future research could accumulate data on more events to develop a predictive model for the certification of care needs based on the findings of this study.

## Supplementary Information

Supplementary Information 1.

Supplementary Table S2.

Supplementary Table S3.

Supplementary Table S4.

Supplementary Table S5.

## Data Availability

The data that support the findings of this study are available from Kitanagoya City but restrictions apply to the availability of these data, which were used under license for the current study, and so are not publicly available. Data are, however, available from the corresponding authors upon reasonable request and with the permission of Kitanagoya City.
