# Supplementary material for: U-shaped link of health checkup data and need for care using a time-dependent cox regression model with a restricted cubic spline
Source: Sci Rep. 2023 May 22;13:7537. doi: 10.1038/s41598-023-33865-x (PMC10203122; doi:10.1038/s41598-023-33865-x)
Supplement: Supplementary file 1 — Supplementary Information 1. [file 41598_2023_33865_MOESM1_ESM.pdf]

U-shaped Link of Health Checkup Data and Need for Care using a Time-dependent Cox Regression Model with a Restricted Cubic Spline

Masahiro Nakatochi, Akitaka Sugishita, Chihiro Watanabe, Etsuko Fuchita, Masaaki Mizuno

SUPPLEMENTARY METHODS

ASSOCIATION OF EACH CONTINUOUS PARAMETER WITH THE EVENTS

For continuous clinical variables, a time-dependent Cox regression analysis[1] was employed to assess the association of each clinical parameter with certified care need and death, using data measured at baseline and over several years of follow-up. Gender and age were included in the model as potential confounders. Furthermore, the same analysis was performed by adding treatment with blood glucose-lowering drugs (yes = 1, no = 0), treatment with antidyslipidemic drugs (yes = 1, no = 0), treatment with antihypertensive drugs (yes = 1, no = 0), smoking status (habitual smoker: yes = 1 vs. no=0), and drinking status (habitual or chance drinker = 1 vs. non-drinker = 0) to the model as potential confounders, in addition to age and gender.

Given that the distribution of triglyceride, ALT, AST, γ-GTP, and HbA1c levels at baseline was skewed (Supplementary Fig. S1), the values were $\log_2$-transformed.

Cox regression analysis was compared by building both linear and nonlinear models. In the case of the linear model, the measured value or $\log_2$-transformed value of each clinical parameter was used as the independent variable. In the linear model, the Cox regression model was fitted as follows:

$$h\big(t, \boldsymbol{x}(t)\big) = h_0(t) \exp\big[\beta_{\text{sex}} x_{\text{sex}} + \beta_{\text{age}} x_{\text{age}}(t) + \textstyle\sum_i \beta_i x_i(t) + \beta_{\text{cp}} x_{\text{cp}}(t)\big],$$

where $\boldsymbol{x}(t)$ is the entire collection of independent variables at time *t*, and $h\big(t, \boldsymbol{x}(t)\big)$ is the hazard at time *t* for a subject with a given specification of $\boldsymbol{x}(t)$. $h_0(t)$ is the baseline

hazard. x_{sex} is a sex and time-fixed variable. The male is coded as 1 and the female is coded as 0. $x_{\text{age}}(t)$ is the age at time *t*. $x_i(t)$ is the value of each of the other covariates at time *t*. $x_{\text{cp}}(t)$ is the value of the clinical parameter at time *t*. $x_{\text{age}}(t)$, $x_i(t)$ and $x_{\text{cp}(t)}$ are time-dependent variables. β_{sex}, β_{age}, β_i, and β_{cp} are regression coefficients for sex, age, other covariates, and clinical parameter, respectively.

In the spline model, the Cox regression model was fitted as follows:

$$h(t, \boldsymbol{x}(t)) = h_0(t) \exp\left[\beta_{\text{sex}} x_{\text{sex}} + \beta_{\text{age}} x_{\text{age}}(t) + \textstyle\sum_i \beta_i x_i(t) + f\left(x_{\text{cp}}(t)\right)\right],$$

where $f\left(x_{\text{cp}}(t)\right)$ is the restricted cubic spline function of $x_{\text{cp}}(t)$ with three knots k_1, k_2, and k_3. $f\left(x_{\text{cp}}(t)\right)$ is given by

$$f\left(x_{\text{cp}}(t)\right) = \beta_{\text{cp1}} x_{\text{cp}}(t)$$

$$+\beta_{\text{cp2}} \left\{\left(x_{\text{cp}}(t) - k_1\right)_+^3 - \left(x_{\text{cp}}(t) - k_2\right)_+^3 (k_3 - k_1)/(k_3 - k_2) + \left(x_{\text{cp}}(t) - k_3\right)_+^3 (k_2 - k_1)/(k_3 - k_2)\right\}$$

$$/(k_3 - k_1)^2,$$

where

$$(u)_+ = u, u > 0,$$

$$0, u \leq 0.$$

Statistical significance in the linear model was tested by comparing the linear model to the model including only the covariates (null model), using likelihood ratio tests. In the case of the non-linear model, the restricted cubic spline was applied to the time-dependent Cox regression model with the adjustment for age and gender to assess the relationship between the continuous variables and adjusted hazard ratio for certification or death. The number of knots was set to three because we wanted to evaluate a U-shaped association between clinical variables and events. Knots were placed at the 10th, 50th, and 90th percentiles of each clinical parameter at baseline.[2] Statistical significance in the nonlinear model was tested by performing a likelihood ratio test comparing the spline and null models.

Statistical significance for non-linearity was tested by performing a likelihood ratio test comparing the spline and linear models. A p-value < .05 for the test of nonlinearity and a p-value < .05 for the spline model depict a statistically significant nonlinear relationship between the clinical parameter and the event, and a spline model is adopted. Conversely, a linear model is adopted for a p-value ≥ .05 for the test of nonlinearity and a p-value < .05 for the linear model. Values of the Akaike information criterion were compared between linear and nonlinear models to assess the quality of each model. To test the proportional hazard assumption of each model, we used the scaled Schoenfeld method.

All analyses were performed using R, version 3.6 (https://www.r-project.org/). A p-value of < .05 was considered significant. We used the survival package[3] and rms package[2] for the time-dependent Cox analysis with a restricted cubic spline. To plot the scaled Schoenfeld residuals, we used the survminer package.

REFERENCES

1 Fisher, L. D. & Lin, D. Y. Time-dependent covariates in the Cox proportional-hazards regression model. *Annu. Rev. Public Health* **20**, 145-157; 10.1146/annurev.publhealth.20.1.145, PMID: 10352854 (1999).

2 Harrell, F. E. Regression modeling strategies 2nd Ed., (Springer, 2015).

3 Therneau, T. M. & Grambsch, P. M. Modeling survival data: extending the Cox model. (Springer, 2010).

Supplementary Fig. S1. Histograms of the continuous clinical variables.

a. BMI

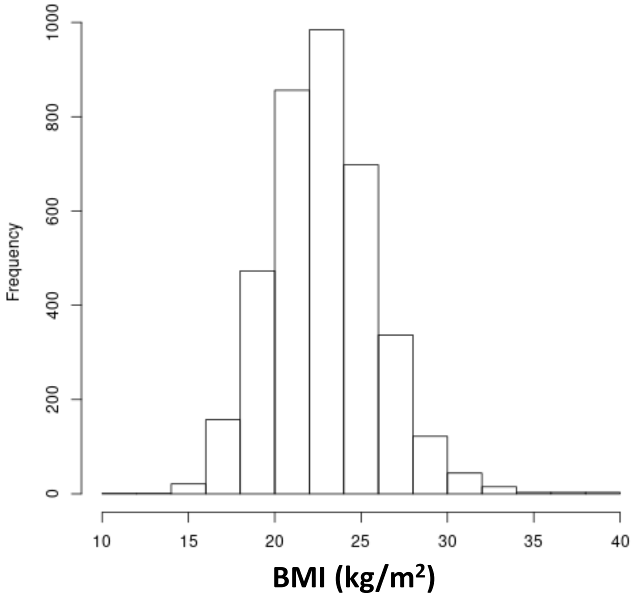

b. SBP

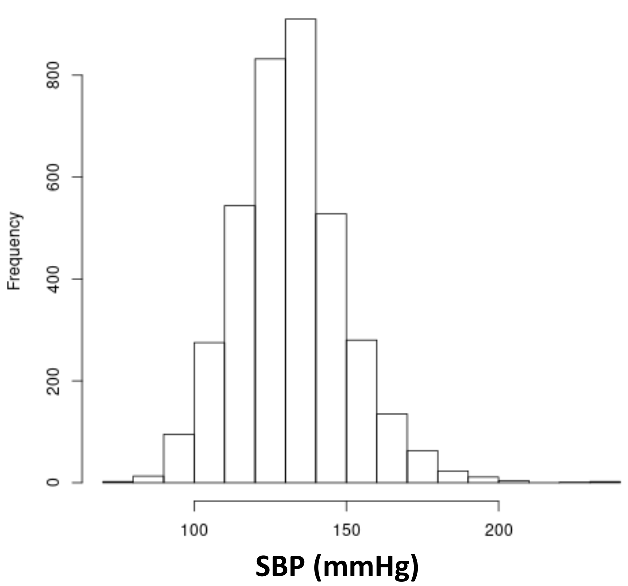

c. DBP

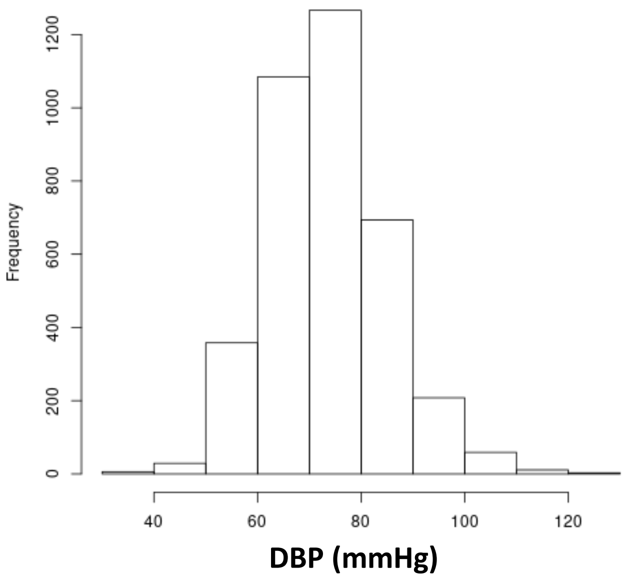

d. HDL-cho

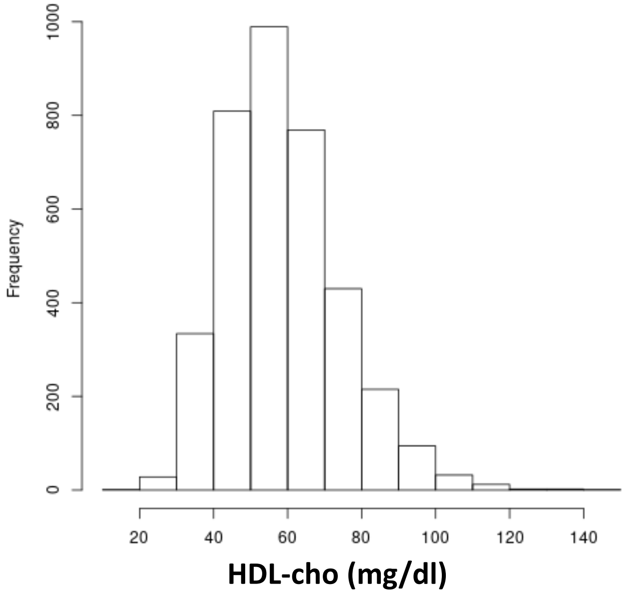

e. LDL-cho

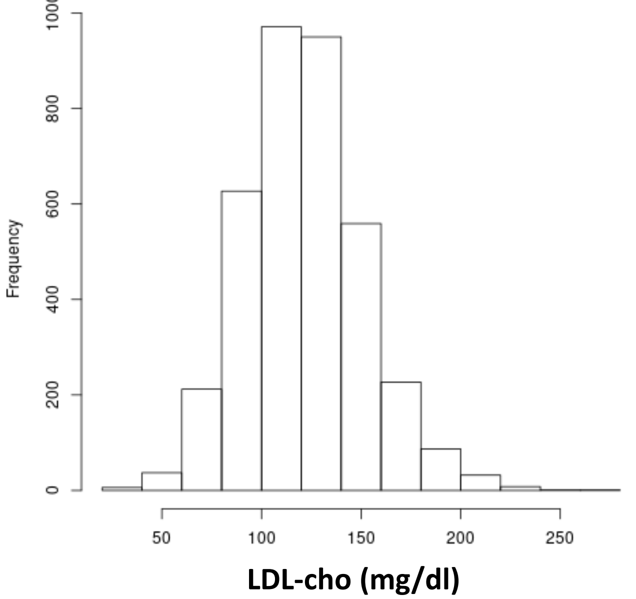

f. Triglyceride

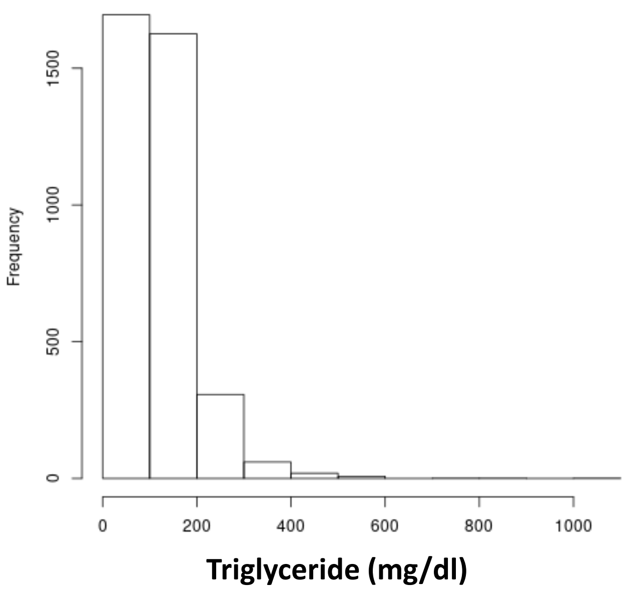

Supplementary Fig. S1. (continued)

g. AST

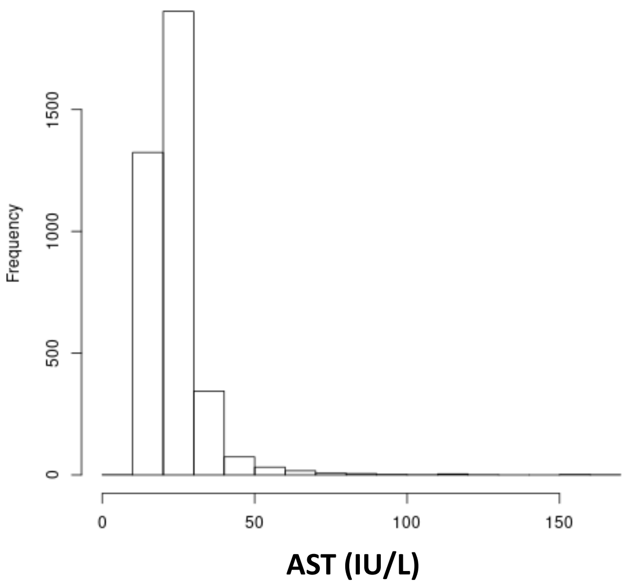

h. ALT

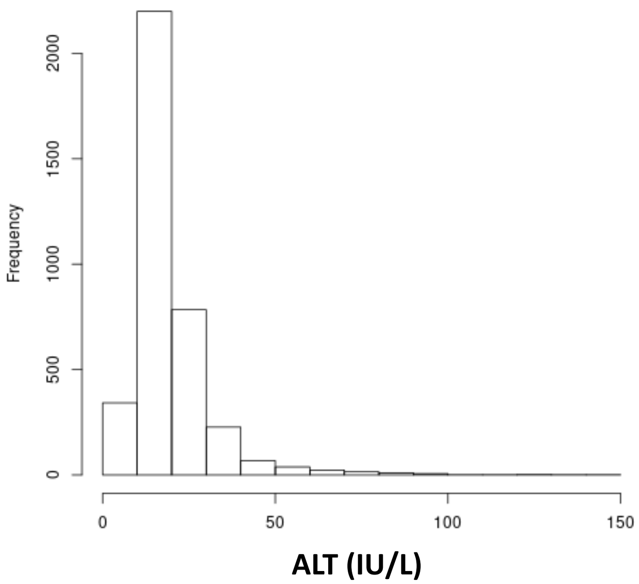

i. γ-GTP

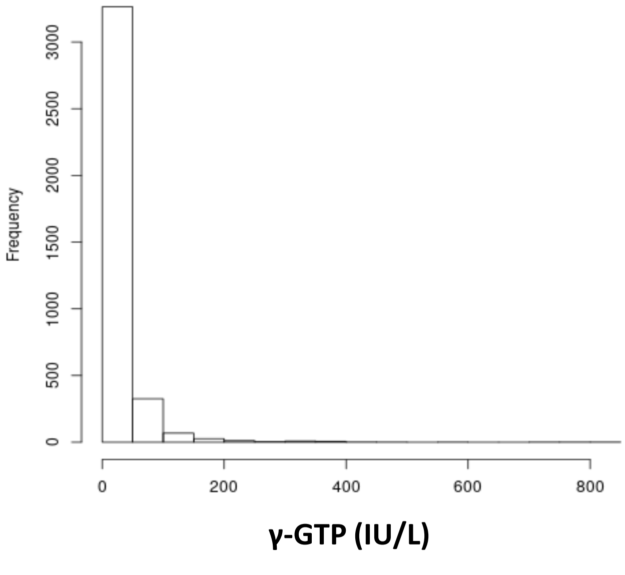

j. HbA1c

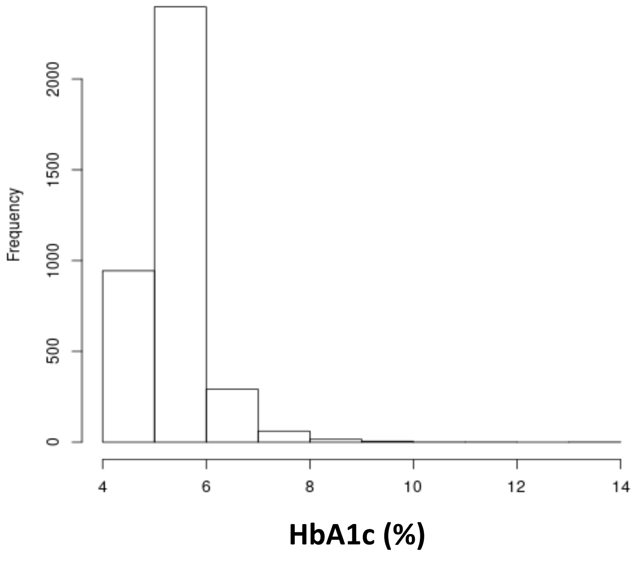

BMI: body mass index; SBP: systolic blood pressure; DBP: diastolic blood pressure; HDL-cho: high-density lipoprotein cholesterol; LDL-cho: low-density lipoprotein cholesterol; AST: aspartate aminotransferase; ALT: alanine aminotransferase; γ-GTP: γ-glutamyl transpeptidase

Supplementary Fig. S2. Testing the proportional hazard assumption in Cox models for the need for care.

a. BMI: spline model

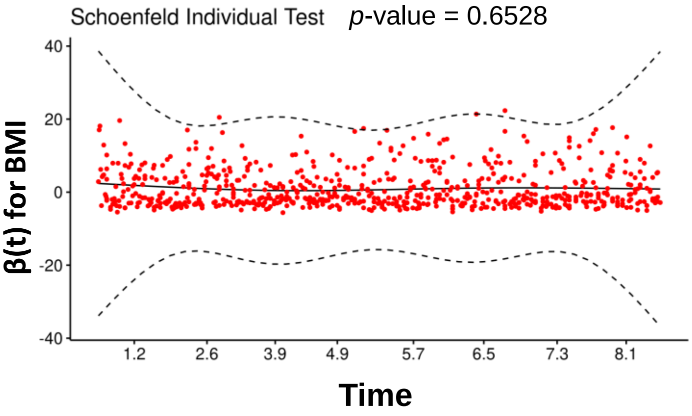

b. SBP: spline model

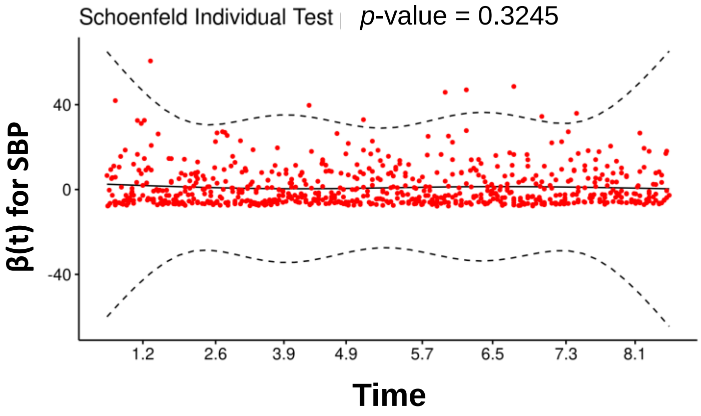

c. HDL-cho: spline model

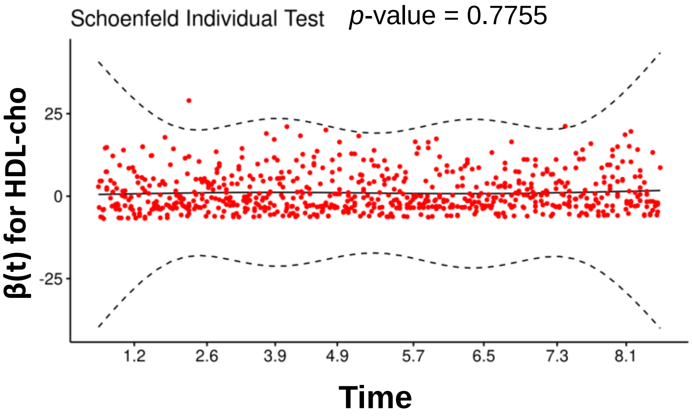

d. LDL-cho: linear model

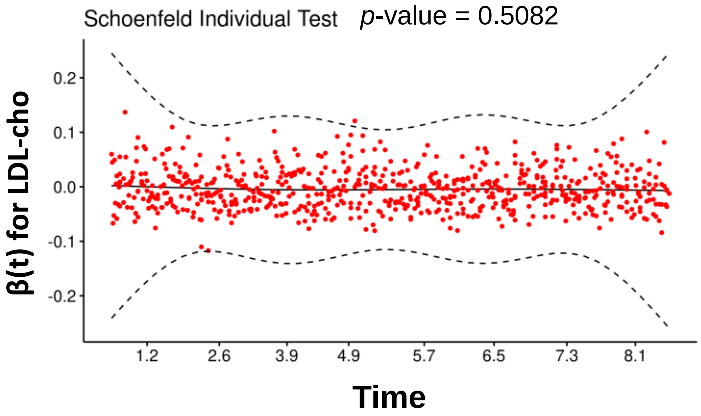

e. $\log_2$(AST): spline model

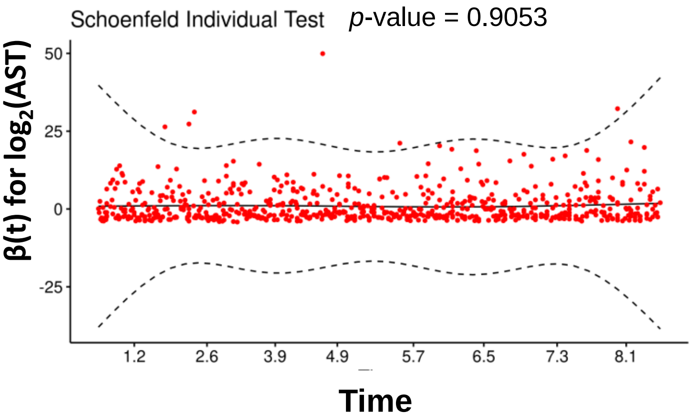

f. $\log_2$(ALT): spline model

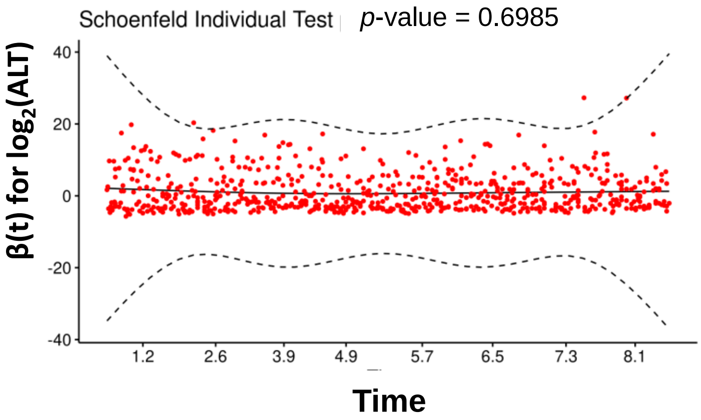

g. $\log_2$(γ-GTP): spline model

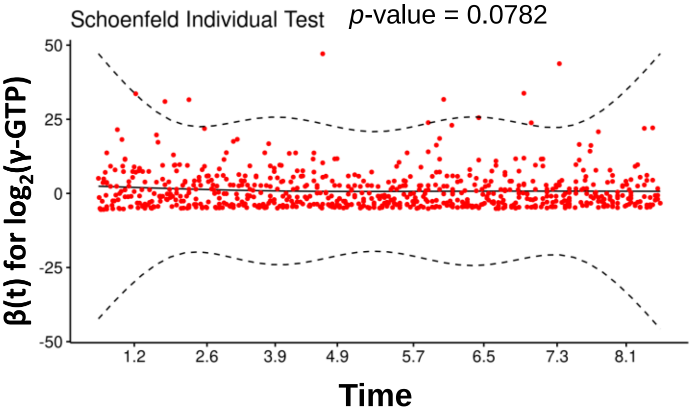

h. $\log_2$(HbA1c): linear model

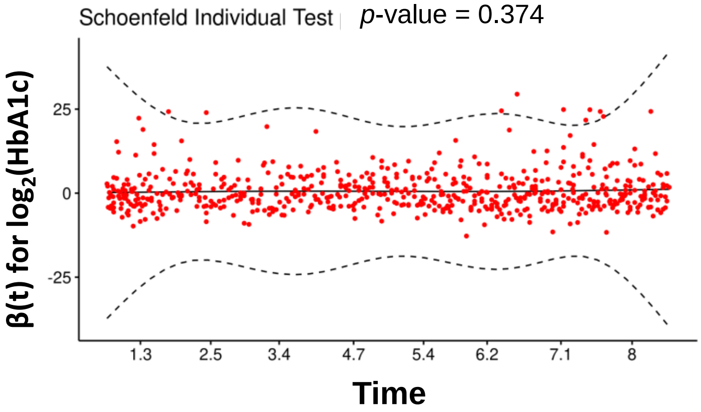

BMI: body mass index; SBP: systolic blood pressure; HDL-cho: high-density lipoprotein cholesterol; LDL-cho: low-density lipoprotein cholesterol; AST: aspartate aminotransferase; ALT: alanine aminotransferase; γ-GTP: γ-glutamyl transpeptidase

Supplementary Fig. S3. Testing the proportional hazard assumption in Cox models for all-cause death.

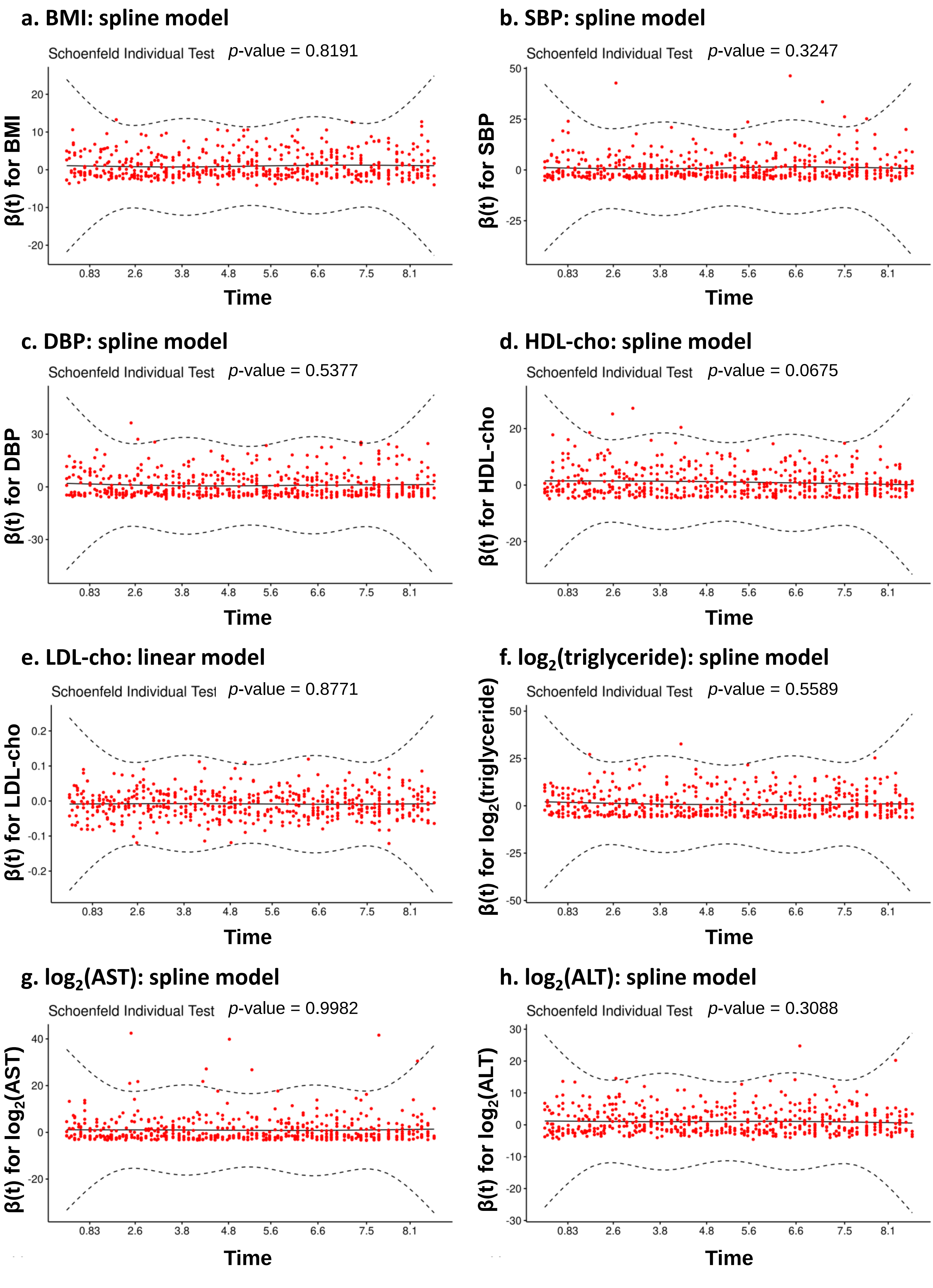

Supplementary Fig. S5. (continued)

i. $log_2(\gamma\text{-GTP})$: spline model

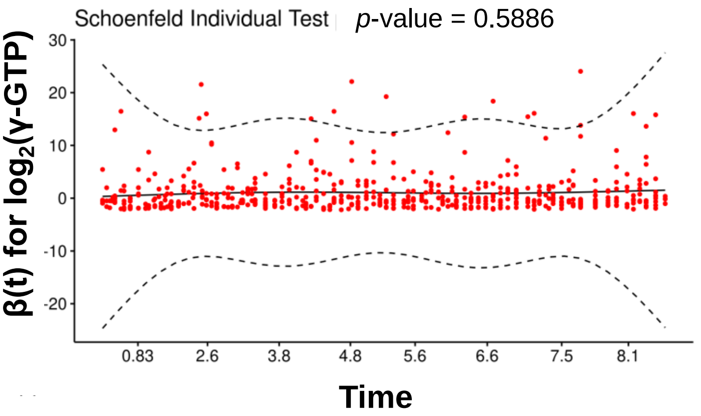

j. HbA1c: linear model

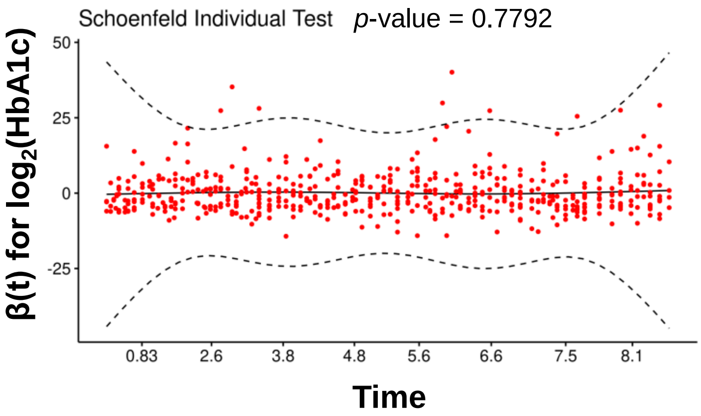

BMI: body mass index; SBP: systolic blood pressure; DBP: diastolic blood pressure; HDL-cho: high-density lipoprotein cholesterol; LDL-cho: low-density lipoprotein cholesterol; AST: aspartate aminotransferase; ALT: alanine aminotransferase; γ-GTP: γ-glutamyl transpeptidase

Supplementary Fig. S4. Hazard ratios of certified need for care estimated by a time-dependent Cox model with a restricted cubic spline model (three knots) adjusted for lifestyle and treatment.
The black straight line indicates the hazard ratio (HR) estimated for the spline model. The gray area represents the 95% confidence intervals of HR for the spline model. The vertical gray straight line indicates the reference value for the clinical parameter. The reference value was calculated as a median value of the clinical parameter. The dot-dash line indicates the HR estimated for the linear model. HRs are estimated when age is fixed at median age at baseline and gender is fixed at female. The *p*-values shown in the figures were calculated by the likelihood ratio test of the spline model against the null model. All *p*-values for nonlinearity were less than 0.05.

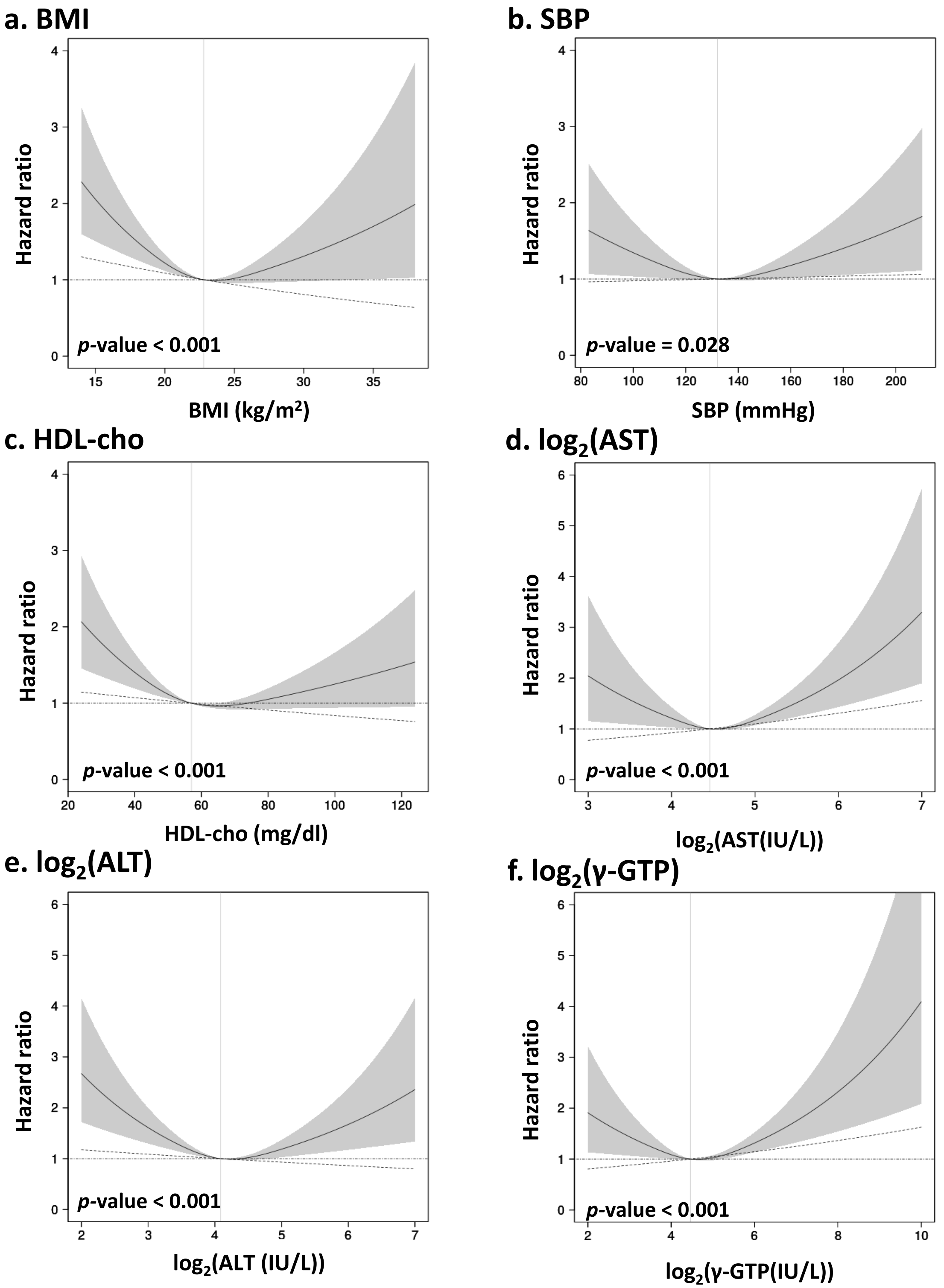

BMI: body mass index; SBP: systolic blood pressure; HDL-cho: high-density lipoprotein cholesterol; AST: aspartate aminotransferase; ALT: alanine aminotransferase; γ-GTP: γ-glutamyl transpeptidase

Supplementary Fig. S5. Hazard ratios for all-cause deaths estimated by a time-dependent Cox model with a restricted cubic spline model (three knots) adjusted for lifestyle and treatment.
The black straight line indicates the hazard ratio (HR) estimated for the spline model. The gray area represents the 95% confidence intervals of HR for the spline model. The vertical gray straight line indicates the reference value for the clinical parameter. The reference value was calculated as a median value of the clinical parameter. The dot-dash line indicates the HR estimated for the linear model. HRs are estimated when age is fixed at median age at baseline and gender is fixed at female. The *p*-values shown in the figures were calculated by the likelihood ratio test of the spline model against the null model. All *p*-values for nonlinearity were less than 0.05.

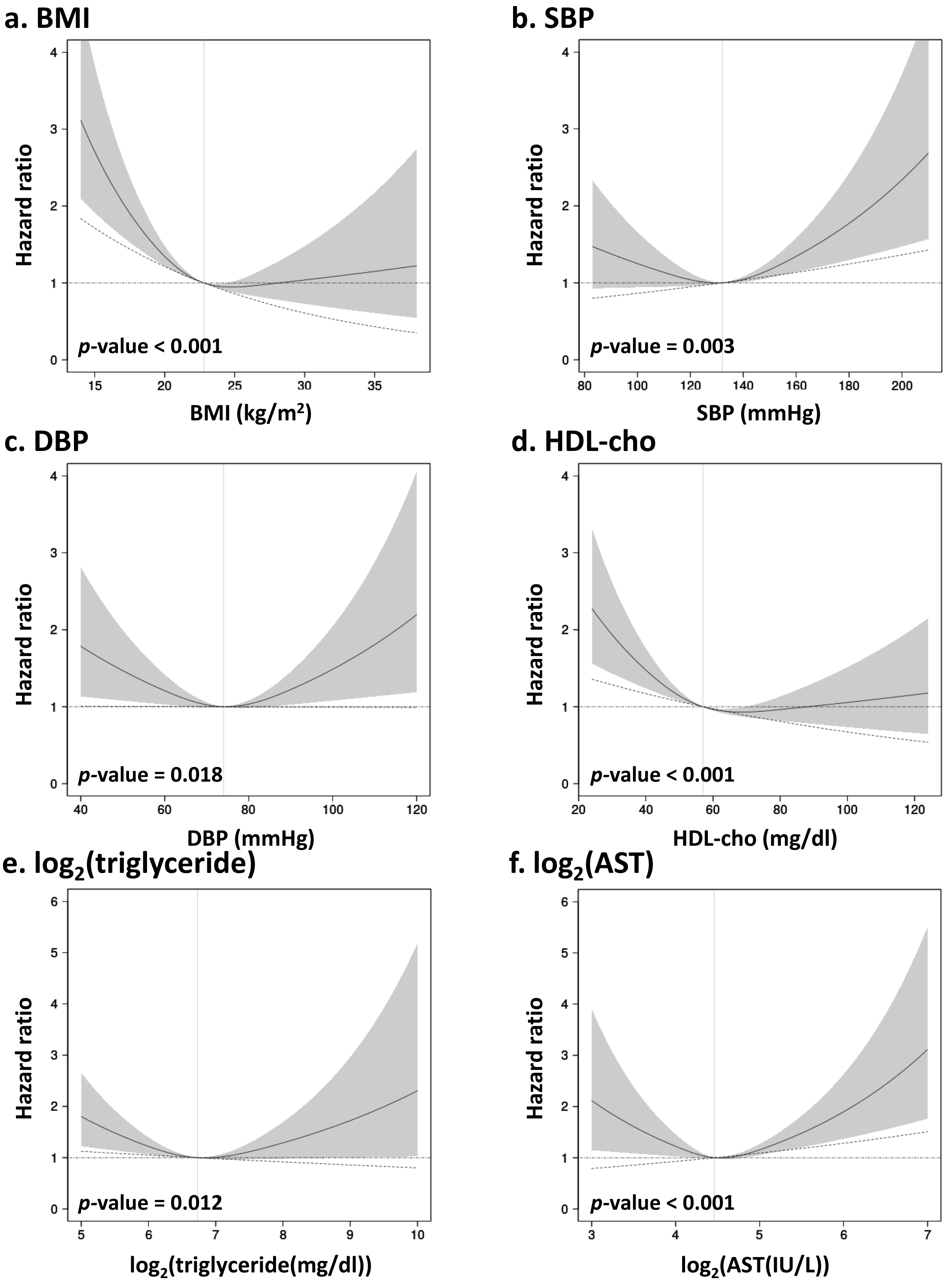

Supplementary Fig. S5. (continued)

g. $\log_2$(ALT)

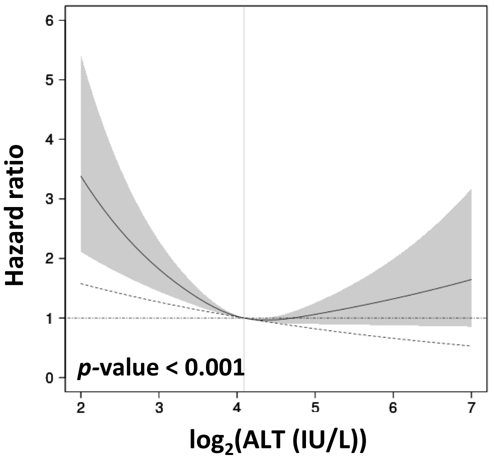

h. $\log_2$(γ-GTP)

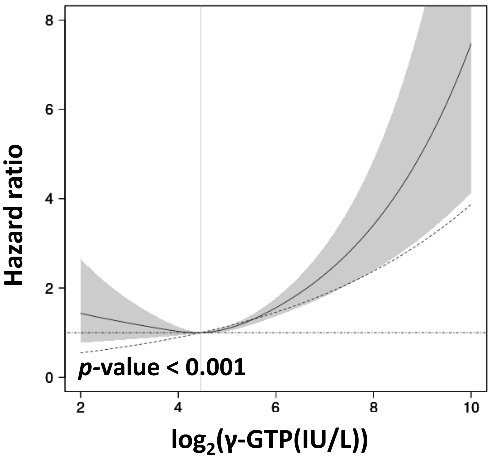

BMI: body mass index; SBP: systolic blood pressure; DBP: diastolic blood pressure; HDL-cho: high-density lipoprotein cholesterol; LDL-cho: low-density lipoprotein cholesterol; AST: aspartate aminotransferase; ALT: alanine aminotransferase; γ-GTP: γ-glutamyl transpeptidase

Supplementary Fig. S6. Testing the proportional hazard assumption in Cox models for the need for care, adjusted for lifestyle and treatment.

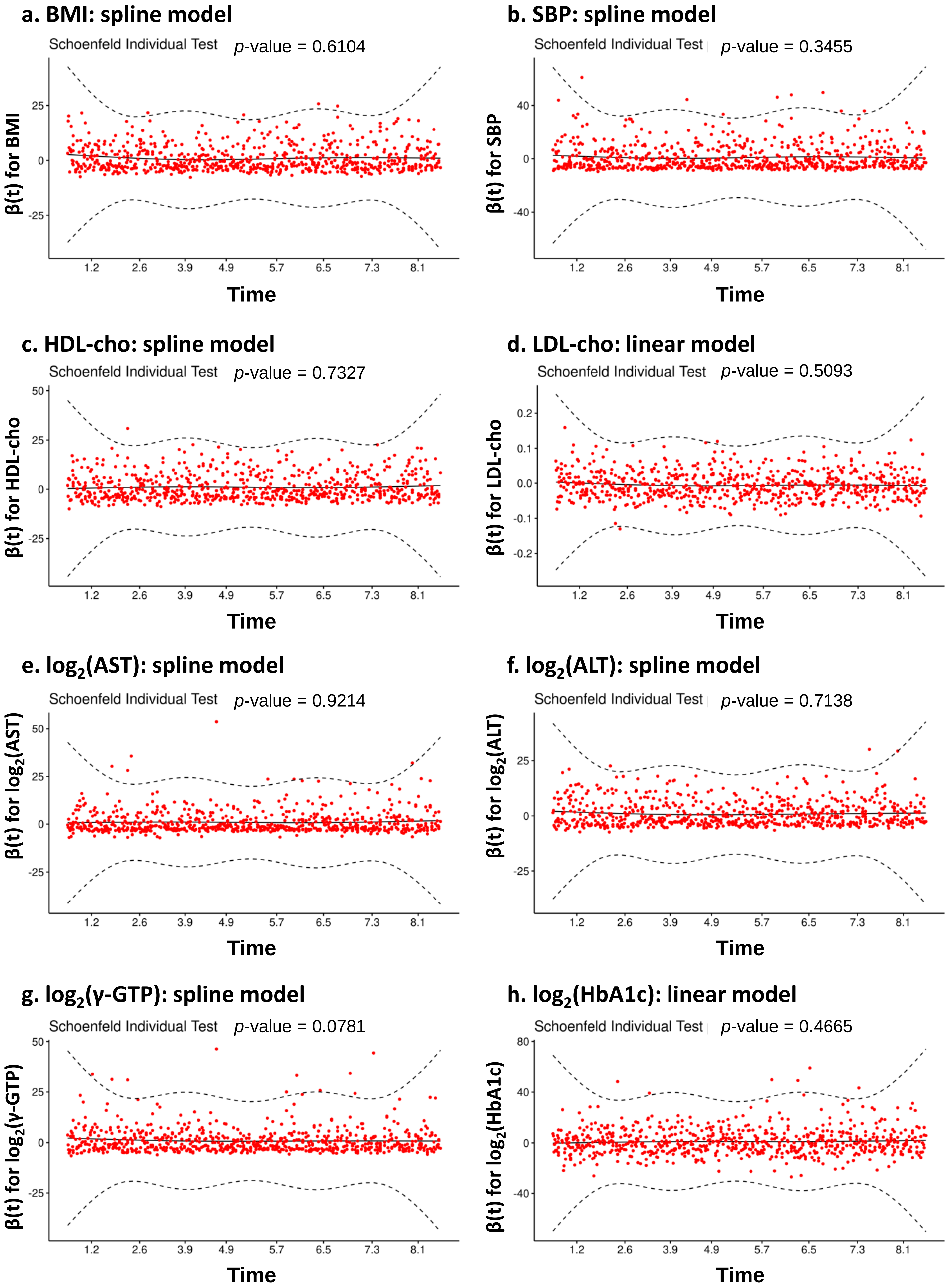

BMI: body mass index; SBP: systolic blood pressure; HDL-cho: high-density lipoprotein cholesterol; LDL-cho: low-density lipoprotein cholesterol; AST: aspartate aminotransferase; ALT: alanine aminotransferase; γ-GTP: γ-glutamyl transpeptidase

Supplementary Fig. S7. Testing the proportional hazard assumption in Cox models for all-cause deaths adjusted for lifestyle and treatment.

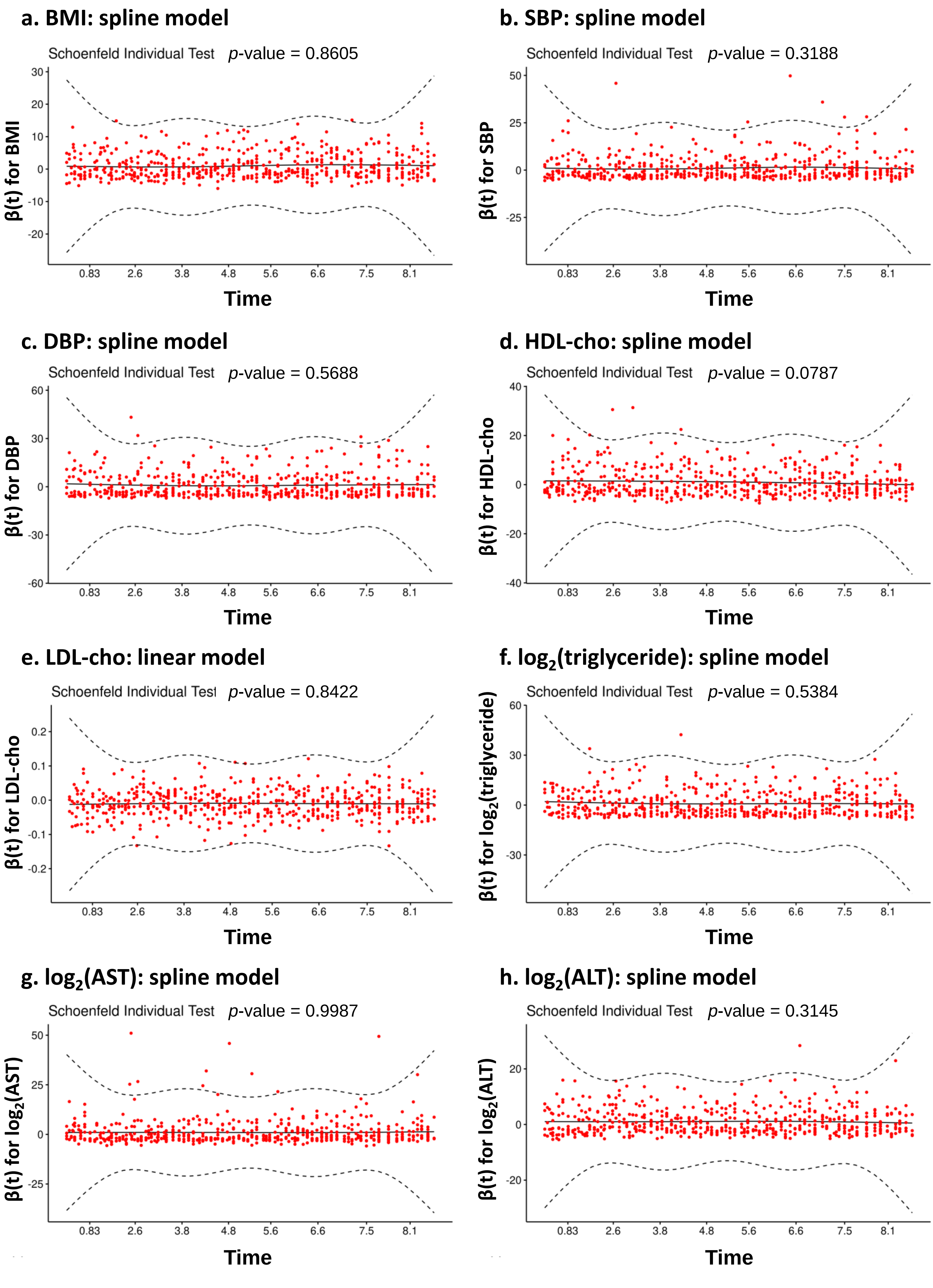

Supplementary Fig. S7. (continued)

i. $\log_2$(γ-GTP): spline model

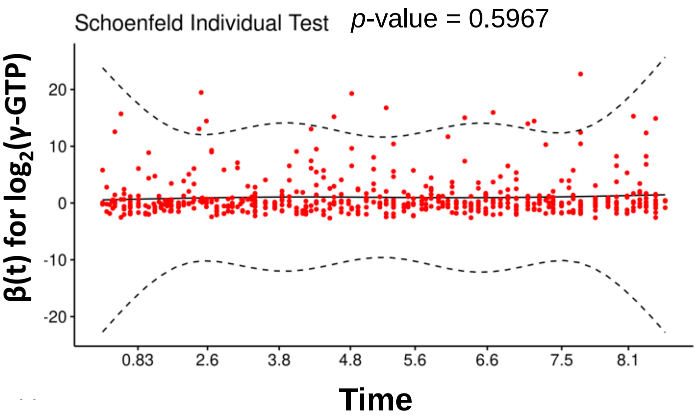

j. HbA1c: linear model

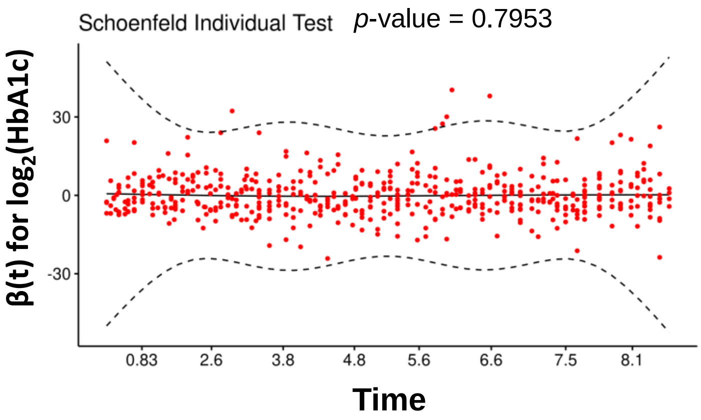

BMI: body mass index; SBP: systolic blood pressure; DBP: diastolic blood pressure; HDL-cho: high-density lipoprotein cholesterol; LDL-cho: low-density lipoprotein cholesterol; AST: aspartate aminotransferase; ALT: alanine aminotransferase; γ-GTP: γ-glutamyl transpeptidase

Supplementary Table S1. Characteristics of participants for categorical variables at the baseline

Variable	Category	N (%)		*p*-value
		**Male**	**Female**	
Age group	65–69	588 (33.8%)	720 (36.4%)	0.212
	70–74	622 (35.7%)	651 (32.9%)	
	75–79	339 (19.5%)	384 (19.4%)	
	80–84	145 (8.3%)	151 (7.6%)	
	85–89	41 (2.4%)	55 (2.8%)	
	≥ 90	7 (0.4%)	15 (0.8%)	
**Urinalysis**				
Urine glucose	-	1513 (87.0%)	1831 (93.1%)	< 0.001
	±	25 (1.4%)	10 (0.5%)	
	+	155 (8.9%)	109 (5.5%)	
	++	18 (1.0%)	5 (0.3%)	
	+++	28 (1.6%)	11 (0.6%)	
Urine protein	-	1339 (77.0%)	1638 (83.3%)	< 0.001
	±	226 (13.0%)	201 (10.2%)	
	+	121 (7.0%)	98 (5.0%)	
	++	35 (2.0%)	22 (1.1%)	
	+++	18 (1.0%)	7 (0.4%)	
**History of disorder**				
Undergoing treatment with blood glucose-lowering drugs	No	1583 (90.9%)	1855 (93.9%)	< 0.001
	Yes	159 (9.1%)	121 (6.1%)	
Undergoing treatment with antidyslipidemic drugs	No	1356 (77.8%)	1336 (67.6%)	< 0.001
	Yes	386 (22.2%)	640 (32.4%)	
	No	917 (52.6%)	1095 (55.4%)	0.093

Undergoing treatment with antihypertensive drugs	Yes	825 (47.4%)	881 (44.6%)	
History of stroke	No	1625 (93.3%)	1902 (96.3%)	< 0.001
	Yes	117 (6.7%)	73 (3.7%)	
History of heart disease	No	1538 (88.3%)	1829 (92.7%)	< 0.001
	Yes	204 (11.7%)	145 (7.3%)	
History of renal failure	No	1732 (99.4%)	1965 (99.5%)	0.825
	Yes	10 (0.6%)	10 (0.5%)	
History of anemia	No	1623 (93.2%)	1771 (89.9%)	< 0.001
	Yes	119 (6.8%)	199 (10.1%)	
**Disorder**				
Underweight	No	1658 (95.2%)	1786 (90.4%)	< 0.001
	Yes	84 (4.8%)	190 (9.6%)	
Overweight or obese	No	1304 (74.9%)	1556 (78.7%)	0.006
	Yes	438 (25.1%)	420 (21.3%)	
Diabetes	No	1488 (85.4%)	1761 (89.1%)	< 0.001
	Yes	254 (14.6%)	215 (10.9%)	
Hypertension	No	656 (37.7%)	784 (39.7%)	0.212
	Yes	1086 (62.3%)	1192 (60.3%)	
Dyslipidemia	No	733 (42.1%)	680 (34.4%)	< 0.001
	Yes	1009 (57.9%)	1296 (65.6%)	
**Lifestyle**				
Habitual smoker	No	1445 (83.0%)	1918 (97.1%)	< 0.001
	Yes	297 (17.0%)	58 (2.9%)	
Drinker	Non	623 (35.8%)	1505 (76.3%)	< 0.001
	Chance	419 (24.1%)	241 (17.8%)	
	Habitual	697 (40.1%)	116 (5.9%)	

The *p*-values were calculated using Fisher's exact test.
